# Supplementary material for: Inference of malaria reproduction numbers in three elimination settings by combining temporal data and distance metrics
Source: Sci Rep. 2021 Jul 14;11:14495. doi: 10.1038/s41598-021-93238-0 (PMC8280212; doi:10.1038/s41598-021-93238-0)
Supplement: Supplementary file 1 — Supplementary Information 1. [file 41598_2021_93238_MOESM1_ESM.docx]

**Supplementary Information for:**

**“Inference of malaria reproduction numbers in three elimination settings by combining temporal data and distance metrics ”**

**Isobel Routledge, H. Juliette T. Unwin, Samir Bhatt**

**Supplementary Table 1:** Full results of ΔAICc and Akaike Weights for each scenario, dataset and spatial kernel considered

**Supplementary Text 1:** Testing approach on simulated data

**Supplementary Figure 1:** A) Plot showing relationship between distance and probability density of two cases being linked by transmission used in simulation and the prior set for inference algorithm.

B) Plot showing relationship between time and probability density of two cases being linked by transmission used in simulation and the prior set for inference algorithm.

**Supplementary Text 1: Testing approach on simulated data**

To “sanity check” our method, we created a simple simulator to generate toy example line lists, as detailed below, and test whether the correct Rc values are estimated when the mean of the hyperparameter defining the prior distribution is the same as the “true” value (see Figure 1). The simulated line list data generated can be found at <https://github.com/IzzyRou/spatial_rcs/tree/main/sim_linelist>

1. We set a number of seed infectors, $j$(Generation 0, G0) which can go on to infect others. Each seed is associated with a time (set at 0 for G0) and a distance ID (randomly generated between 0 and 10 000). The number of individuals they will go onto infect ($i$) is drawn from a poisson distribution, with a mean reproduction number.

$N\left( Infections leading to G1 \right)\sim Poisson(0.5)$ (equation 1)

1. These seed infections lead to Generation 1 (G1). To simulate the times of infection ($T_{i}$ for generation 1, and their distance from infectors in Generation 0 ($D_{i}$, we draw time differences ($T_{i}-T_{j}$) from a normal distribution of mean 40, standard deviation 15. We draw distances $(D_{i}-D_{j})$ from an exponential distribution of rate 0.01.

$$T_{i}-T_{j} \sim Rayleigh\left( 0.01 \right)$$

 (equation 2)$D_{i}-D_{j} \sim Exponential(0.01)$ (equation 3)

1. We then repeat the process, drawing  the number of individuals each individual in G1 will go onto infect  from a poisson distribution, with a mean reproduction number, and drawing time and distance differences from their infectors in the previous generation.

$N\left( Infections leading to G2 \right)\sim Poisson\left( 0.5 \right)$ (equation 4)

1. This is repeated for several generations.
2. From the time and location data, time matrices and distance matrices are created. These are incorporated into the model, with priors set at the “correct” mean value and the mean $R_{c}$ estimate is compared to the “true” mean $R_{c}$ in the data. We did not simulate missing data for this toy example, so the  epsilon parameter was set at a very low value (1e-20).

| **Simulation and inference algorithm parameters** | **True** $R_{c}$ | **Summary of median posterior**$R_{c}$ **estimated from running the algorithm on 10 line-lists generated from simulation** |
| --- | --- | --- |
| $\boldsymbol{N}\left( \boldsymbol{Seed Infections} \right)\boldsymbol{=300}$  $Prior\left( \alpha\right)\sim normal(0.002,0.001)$  $Prior\left( \beta\right)\sim normal(0.01,0.001)$  $\boldsymbol{\varepsilon=1}\boldsymbol{e}^{\boldsymbol{-20}}$ | **0.5** | Median = 0.52  Mean =  0.52  97.5% quantile interval = (0.48, 0.57) |


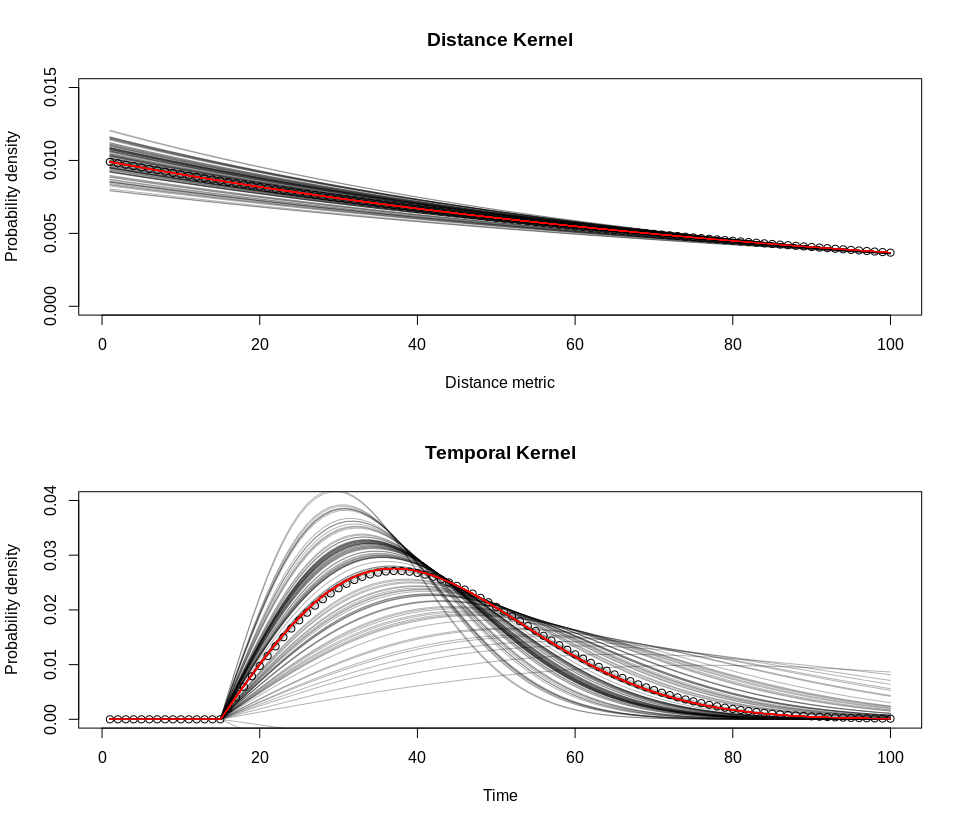


**B**

**A**

**Supplementary Figure 1:** A) Plot showing relationship between distance and probability density of two cases being linked by transmission. Black lines show 100 draws from prior distribution of parameter, shaping the exponential $,Prior\left( \beta\right)\sim normal\left( 0.01,0.001 \right).$ Red line is expectation of the prior, points show the function used in simulation.

B) Plot showing relationship between time and probability density of two cases being linked by transmission. Black lines show 100 draws from prior distribution of parameter alpha, shaping the shifted Rayleigh distribution, $Prior\left( \alpha\right)\sim normal\left( 0.002,0.001 \right).$Red line is expectation of the prior, points show the “true” function used in simulation.
